# Assessing the knowledge, attitudes, and perceptions of ovarian cryopreservation technology among women with ovarian diseases

**DOI:** 10.3389/fpubh.2025.1612492

**Published:** 2025-06-23

**Authors:** Faten Mabrouk Nouh, Hasan Abualruz, Amal Khalifa Khalil, Rania Ezzat El-Gobashy, A. M. F. Alasser, Aya Ibrahim Shaban Abdullah, Jebril Al Hrinat, Aseel Ghaleb Hendi, Majdi AL-Zoubi, Tahani Al Rahbeni, Khalid Al-Mugheed, Sally Mohammed Farghaly Abdelaliem, Hanaa Elsayed Ahmed Shahin

**Affiliations:** ^1^Faculty of Nursing, Al-Zaytoonah University of Jordan, Amman, Jordan; ^2^Maternity and Newborn Health Nursing, Faculty of Nursing, Menoufia University, Menoufia, Egypt; ^3^Faculty of Education, Arab American University, Jenin, Palestine; ^4^The Hashemite University, Amman, Jordan; ^5^Molecular Toxicology and Genetics/College of Pharmacy, Nursing, and Medical Science, Riyadh Elm University, Riyadh, Saudi Arabia; ^6^College of Nursing, Riyadh Elm University, Riyadh, Saudi Arabia; ^7^Department of Nursing Management and Education, College of Nursing, Princess Nourah bint Abdulrahman University, Riyadh, Saudi Arabia; ^8^Department of Nursing, Applied Medical Sciences, Jouf University, Sakakah, Saudi Arabia

**Keywords:** knowledge, attitude, perception, oocyte cryopreservation, ovarian diseases

## Abstract

**Background:**

Cryopreservation techniques for laboratory oocytes provide women with increased reproductive options, especially for those facing fertility challenges due to ovarian diseases.

**Aim:**

This study aimed to assess the knowledge, attitudes, and perceptions of oocyte cryopreservation (OC) technology among women with ovarian diseases.

**Design:**

A cross-sectional, quantitative study was conducted involving 202 women, aged 18–45, who were diagnosed with ovarian diseases. Participants were recruited from outpatient obstetrics and gynecology clinics at the Menoufia University Hospital and Shebin El-Kom Teaching Hospital. Data collection instruments included an online questionnaire, obstetric and gynecological history forms, and structured assessments of knowledge, perceptions, and attitudes toward oocyte cryopreservation.

**Results:**

The findings revealed that 59.9% of the women had a moderate level of knowledge regarding oocyte cryopreservation. More than half (55.9%) demonstrated a negative attitude toward the technology, which significantly influenced their perceptions of it. In addition, a notable proportion of the participants reported experiencing infertility due to their ovarian condition. Among them, 37.6% stated that their disease had a substantial negative impact on their overall quality of life.

**Conclusion:**

The study revealed that a considerable proportion of women with ovarian diseases possessed inadequate knowledge of and had negative attitudes toward oocyte cryopreservation, which adversely affected their perceptions of the procedure. Furthermore, ovarian diseases were found to contribute to infertility and a reduced quality of life. These findings underscore the need for targeted health education programs to improve awareness of, attitudes toward, and understanding of fertility preservation options in this population.

## Introduction

Oocyte cryopreservation (OC), commonly known as egg freezing, is a reproductive technology that involves freezing and storing a woman’s eggs for future use in assisted reproductive treatments, such as *in vitro* fertilization (IVF) (1). Ovarian tissue cryopreservation (OTC), once considered experimental, is now increasingly recognized—particularly by the American Society for Reproductive Medicine—as a valid fertility preservation method for women who cannot delay gonadotoxic treatments and for prepubertal girls (2). OTC allows for the restoration of both fertility and ovarian endocrine function, unlike oocyte cryopreservation alone.

There are two main methods of OTC: one involves freezing the entire ovary, along with its blood supply, for reimplantation, while the other involves freezing only the ovarian cortical tissue for avascular grafting. Over 130 healthy births have been reported globally as a result of OTC, with promising success rates following transplantation (3). The increasing average age of first-time pregnancies—now over 30 years—reflects modern challenges such as career focus, educational commitments, financial instability, and difficulty finding partners (4). As women age, the quantity and quality of their eggs decline, making cryopreservation an increasingly important fertility option. OTC is particularly beneficial for women with conditions such as polycystic ovary syndrome (PCOS), endometriosis, or ovarian cysts, as it helps them avoid premature menopause and its associated health risks (5).

Globally, infertility affects approximately 80 million people, with 10–15% of couples experiencing it at some point in their lives (1). In Egypt, infertility affects 10.4% of married couples, with premature ovarian failure occurring in approximately 8% of women under 40 —a rate significantly higher than the global average of 1% (6). Educating individuals about ovarian diseases and fertility preservation options can lead to earlier diagnoses and improved outcomes. Knowledge and attitudes toward oocyte cryopreservation are essential for effective counseling. Studies indicate that self-assessments of attitudes can reveal underlying assumptions about the acceptability and feasibility of this procedure, especially among women of childbearing age who have a history of gynecological issues (7, 8).

Despite the proven effectiveness of cryopreservation in assisted reproduction, many studies have shown limited awareness and understanding of the procedure among women and healthcare students. For example, while ovarian tissue cryopreservation has preserved fertility in cancer patients for more than 20 years (9), many are unaware of this procedure or hold misconceptions about it. Premature ovarian insufficiency (POI), which is often caused by cancer treatments or genetic and autoimmune factors, emphasizes the need for fertility preservation options (10). Research conducted by medical and nursing students has revealed that there is limited knowledge of and willingness to undergo elective oocyte cryopreservation (11, 12). Several studies have emphasized the need to incorporate fertility preservation education into medical curricula to equip future healthcare providers with the knowledge necessary for effective patient counseling (8, 13). While attitudes toward elective cryopreservation are generally positive, many still lack a clear understanding of the process (7, 14). Although cryopreservation offers numerous benefits, its acceptance is influenced by awareness, attitudes, and perceptions. Healthcare professionals must assess and address the knowledge and beliefs of women—particularly those with ovarian disease—to promote informed decision-making. This study seeks to explore three key areas:

1. What is the level of knowledge among women with ovarian diseases regarding oocyte cryopreservation?
2. What are their attitudes toward this technology?
3. What are their perceptions of its use and value?

## Materials and Methods

### Research Design

This study utilized a descriptive cross-sectional research design.

### Participants

A total of 202 women diagnosed with ovarian disease were recruited from the outpatient clinics at Menoufia University and Shebin El-Kom Teaching Hospitals to participate in this study. A purposive sampling technique was employed to include women aged 18–45 who had received information or consultation regarding ovarian cryopreservation technology as part of their fertility treatment plan. All participants provided their informed consent. However, it is important to note that the selection criteria did not fully align with the research objectives. While the study aimed to assess the aspects related to ovarian cryopreservation, the inclusion of participants solely based on an ovarian disease diagnosis—without verifying whether they were specifically affected by diminished ovarian reserve or other egg-related fertility-threatening conditions—may have resulted in a sample that does not accurately represent the intended target population. Women without a diagnosis of ovarian disease, those unwilling to participate, those not informed about ovarian cryopreservation, and those with cognitive impairments that could affect comprehension and consent were excluded from the study.

### Data collection tools

An online questionnaire was designed by the researchers after reviewing the relevant literature from 1 July 2024 to 1 September 2024. The questionnaire, administered in Arabic, was divided into four sections:

*Section I:* Online questionnaire: This was developed by the researcher based on a review of recent related literature (15–20). It included three parts.

- *Part one:* Sociodemographic characteristics of women with ovarian diseases.
- *Part two:* Medical history of women with ovarian diseases, including the type of ovarian disease, symptoms experienced, time of diagnosis, treatment received for fertility issues related to the ovarian disease, and any advice given to undergo any fertility treatments or interventions.
- *Part three:* The effect of ovarian diseases on the reproductive health of women, including difficulty conceiving, irregular menstrual cycles, pain during menstruation or intercourse, and early menopause.
  *Section II:* Assessment of the knowledge of women with ovarian diseases regarding oocyte cryopreservation, based on recent relevant literature (18, 19). This section covered topics such as the definition, methods, indications, advantages, disadvantages, general success rates, cost, duration of storage, regulations and storage procedures, practice guidelines, and infection control.
  *Section III:* Assessment of the attitudes of women with ovarian diseases toward oocyte cryopreservation, based on existing literature (15–19, 21). This section included 11 statements.
  *Section IV:* Assessment of the perceptions of women with ovarian diseases regarding oocyte cryopreservation, based on recent relevant literature (15–19, 21). This section includes the following statements: OC is forbidden by religion; OC is not accessible; the success rate of OC is very low; OC should be included in premarital counseling and routine healthcare visits; acceptance of financial support from family for the procedure; babies born via OC are normal and socially acceptable; confidence in doctors and laboratory staff involved in storage; cultural influences on decision-making regarding OC; and the high cost of OC as a barrier.

### Construct reliability and validity

The validity of the instruments was determined by three experts: one was from the Obstetrics and Gynecology Department at the Faculty of Medicine, and two professors were from the Maternal and Newborn Health Nursing Department at the Faculty of Nursing. The researchers utilized test–retest reliability to establish the internal consistency of the instruments by administering the tests to the same subjects under similar conditions two or more times. The Cronbach’s alpha values were 0.79 for the knowledge questionnaire, 0.83 for the perceptions scale, and 0.89 for the attitudes scale.

### Data analysis

Data analysis was conducted using the Statistical Package for the Social Sciences (SPSS) version 23. Before analysis, the dataset was cleaned by removing duplicates and correcting error codes. Descriptive statistics, including means and standard deviations, were computed to summarize the sociodemographic and professional characteristics of the participants. Pearson’s correlation coefficient (r) was used to examine the relationships between continuous variables, with statistical significance set at a *p*-value of < 0.05. Group differences based on variables such as age, education level, and disease type were initially assessed using chi-squared tests.

## Results

### Demographic Data

Table 1 presents the sociodemographic characteristics of the studied women. Less than half (49.0%) held a bachelor’s degree, and over one-third (31.2%) had been married for 1–4 years. The majority of the participants (62.4%) resided in urban areas. In terms of financial capacity, less than half (47.0%) reported having sufficient income to cover treatment-related expenses.

**Table 1:** Demographic characteristics of the studied sample (*n* = 202).

Demographics	No.	%
Educational level		
Diploma	80	39.6
Bachelor’s	99	49.0
Postgraduate studies	23	11.4
Marital duration		
<1– < 4 years	63	31.2
5–7 years	49	24.3
8–10 years	35	17.3
> 10 years	55	27.2
Residence		
Urban	126	62.4
Rural	76	37.6
Income		
Not enough to cover treatment costs	64	31.7
Enough to cover treatment costs	95	47.0
Able to cover treatment costs and save money	43	21.3

**Figure 1:**
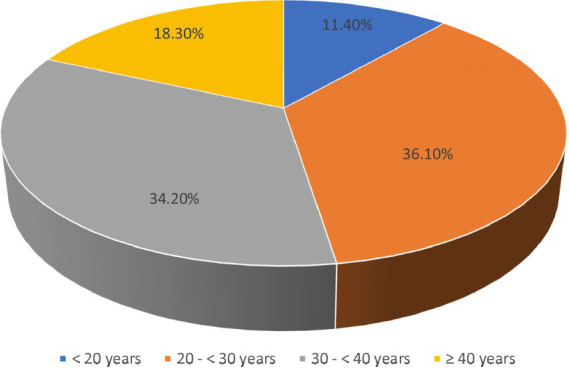
Age of the studied women (*n* = 202).

Figure 1 shows the age distribution of the women included in this study, with over 36.1% falling between the ages of 20 and 30. This highlights the importance of oocyte cryopreservation for younger women dealing with fertility issues related to ovarian diseases.

The results provide a comprehensive overview of the medical history of the group under examination, covering different aspects connected to ovarian conditions. These clearly show that the majority of the women (19.3%) had polycystic ovary syndrome (PCOS). Less than one-quarter (21.8%) of the participants had symptoms of ovarian illness for 1–2 years. Between 1 and 2 years, fewer than one-quarter (21.4%) were diagnosed with ovarian disease. More than two-thirds (70.8%) sought medical treatment for ovarian disease-related reproductive concerns and were encouraged to pursue fertility therapies or interventions (Table 2).

**Table 2:** Medical history of the studied sample (*n* = 202).

Medical history	No.	%
Having conditions that might contribute to ovarian disease		
Polycystic Ovary Syndrome (PCOS)	39	19.3
Endometriosis	20	9.9
Pelvic inflammatory disease (PID)	45	22.3
Ovarian cysts	32	15.8
Hormonal disorders	29	14.4
Uterine polyps or tumors	37	18.3
Duration of symptoms of ovarian disease		
< 6 months	32	15.8
6 months - < 1 year	31	15.3
1–2 years	44	21.8
> 2 years	67	33.2
Not sure	28	13.9
Time of diagnosis		
< 6 months	31	15.4
6 months - < 1 year	35	17.4
1–2 years	43	21.4
> 2 years	64	31.8
Not sure	28	13.9
Seeking medical treatment for fertility issues related to ovarian disease		
Yes	143	70.8
No	59	29.2
Received advice to undergo any fertility treatments or interventions		
Yes	153	76.9
No	46	23.1

**Figure 2:**
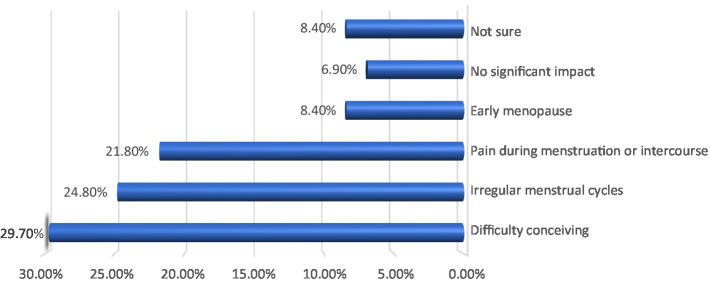
Effect of ovarian disease on the reproductive health of the studied women (*n* = 202).

As for the effect of ovarian disease on the reproductive health of the studied women, the results shown in Figure 2 summarize the negative effects of ovarian diseases on the reproductive health of the women participating in the study (202 in number). The most prominent of these effects was difficulty conceiving, which affected 29.7% of the women, followed by irregular menstrual cycles at 24.8% and pain during menstruation or intercourse at 21.8%. The results also indicated that some women experienced early menopause at 8.4%, while 6.9% of the participants did not observe any noticeable effect on their reproductive health. Thus, ovarian diseases pose a major threat to female fertility and negatively affect the quality of reproductive life for many women.

There was a significant knowledge gap among women regarding egg freezing, as shown in Table 3. Although 40.6% of women were able to correctly define egg freezing, their knowledge regarding the methods used in egg freezing was very limited. Only 13.9% of the participants answered this question correctly. In addition, the results showed that almost half of the women were aware of the benefits of egg freezing; however, a significant percentage were unaware of the total cost of this procedure. In summary, the results shown in this table indicate that more efforts are needed to educate women and raise their awareness regarding egg freezing. This will help them make informed decisions about their reproductive health.

**Table 3:** Women’s knowledge regarding oocyte cryopreservation (*n* = 202).

Items	Correct answer		Incorrect answer		Do not know/Not sure	
	No.	%	No.	%	No.	%
Definition of OC	82	40.6	39	19.3	81	40.1
Methods used for OC	28	13.9	86	42.6	88	43.6
Indications of OC	91	45.0	54	26.7	57	28.2
Advantages of OC	93	46.0	58	28.7	51	25.2
Disadvantages of OC	94	46.5	67	33.2	41	20.3
General success rate of OC	61	30.2	61	30.2	80	39.6
Typical cost of OC	21	10.4	76	37.6	105	52.0
Duration of storing oocytes effectively once frozen	19	9.4	74	36.6	109	54.0
Presence of specific regulations governing the practice of OC	78	38.6	19	9.4	105	52.0
Presence of established practice guidelines for OC	89	44.1	53	26.2	60	29.7

OC, oocyte cryopreservation.

Table 4 illustrates the key perceptions and concerns among the surveyed women regarding oocyte cryopreservation (OC). A significant number of women expressed concerns about the potential for poor storage conditions, prolonged storage duration, and uncertainty regarding the future use of frozen ova. Notably, 36.1% believed that OC is a viable option for unmarried women, and 56.9% supported the promotion of this technology. In addition, a large majority (79.7 and 79.1%, respectively) agreed that ovum freezing banks should be accredited, adhere to standardized protocols, and maintain strict infection control measures during the freezing and storage processes. More than half of the participants indicated they would consider OC for social or career-related reasons, especially if the costs were affordable. Furthermore, 78.7% agreed that ovarian reserve testing should be made widely accessible.

**Table 4:** Women’s attitudes toward oocyte cryopreservation (*n* = 202).

Items	Strongly agree	Agree	Neutral	Disagree	Strongly disagree
	No. (%)	No. (%)	No. (%)	No. (%)	No. (%)
Concern about poor storage of frozen ova	69 (34.2)	96 (47.5)	31 (15.3)	3 (1.5)	3 (1.5)
Concern about the long storage period	64 (31.7)	95 (47.0)	37 (18.3)	3 (1.5)	3 (1.5)
Fear of not using frozen ova in the future	58 (28.7)	78 (38.6)	54 (26.7)	11 (5.4)	1 (0.5)
OC is a viable option for unmarried women	22 (10.9)	51 (25.2)	71 (35.1)	33 (16.3)	25 (12.4)
OC should be encouraged	21 (10.4)	94 (46.5)	66 (32.7)	18 (8.9)	3 (1.5)
Ovum freezing banks should be certified and follow standards	76 (37.6)	85 (42.1)	33 (16.3)	7 (3.5)	1 (0.5)
Freezing and storage operations must be monitored	74 (36.8)	85 (42.3)	34 (16.9)	5 (2.5)	4 (2.0)
Infection control precautions should be used during freezing and storage	84 (41.6)	82 (40.6)	30 (14.9)	3 (1.5)	3 (1.5)
Consideration of OC for social and career reasons	49 (24.3)	71 (35.1)	66 (32.7)	13 (6.4)	3 (1.5)
Consideration of OC if the cost is suitable	54 (26.7)	80 (39.6)	55 (27.2)	11 (5.4)	2 (1.0)
Tests to check ovarian reserve should be freely available	67 (33.2)	91 (45.0)	37 (18.3)	6 (3.0)	1 (0.5)

OC, oocyte cryopreservation.

As shown in Table 5, more than half of the women (54.0%) had a neutral opinion about religious prohibitions regarding OC. Over half (55.4%) concurred that OC is not accessible. Regarding the low success rate of OC, less than half (42.1%) expressed no opinion. The majority of the respondents (55%) felt that OC should be discussed during routine medical checkups and premarital counseling. More than two-thirds (64.3%) indicated that they had accepted financial assistance from their families for the treatment. The vast majority (59.9%) concurred that babies delivered via OC are normal and accepted in society. More than half (51.5%) expressed trust in the doctors and laboratory personnel involved in the storage process. More than two-thirds (69.3%) agreed that cultural factors influence decision-making regarding OC, and more than three-quarters (78.1%) agreed that the high cost of OC is a barrier to its adoption.

**Table 5:** Women’s perceptions about oocyte cryopreservation (*n* = 202).

Items	Strongly agree	Agree	Neutral	Disagree	Strongly disagree
	No. (%)	No. (%)	No. (%)	No. (%)	No. (%)
OC is forbidden by religion	9 (4.5)	14 (6.9)	109 (54.0)	49 (24.3)	21 (10.4)
OC is not accessible	41 (20.3)	71 (35.1)	68 (33.7)	15 (7.4)	7 (3.5)
The success rate of OC is very low	14 (6.9)	65 (32.2)	85 (42.1)	32 (15.8)	6 (3.0)
OC should be included in premarital counseling and routine healthcare visits	24 (11.9)	87 (43.1)	63 (31.2)	19 (9.4)	9 (4.5)
Acceptance of financial support from family for the procedure	32 (15.8)	98 (48.5)	57 (28.2)	13 (6.4)	2 (1.0)
Babies born via OC are normal and socially acceptable	33 (16.3)	88 (43.6)	65 (32.2)	15 (7.4)	1 (0.5)
Confidence in the doctors and laboratory staff involved in the storage process	27 (13.4)	77 (38.1)	76 (37.6)	15 (7.4)	7 (3.5)
Cultural influences on decision-making regarding OC.	47 (23.3)	93 (46.0)	56 (27.7)	5 (2.5)	1 (0.5)
The high cost of OC is a barrier.	80 (39.8)	77 (38.3)	41 (20.4)	2 (1.0)	1 (0.5)

OC, oocyte cryopreservation.

Using Pearson’s correlation coefficient, it was found that the correlation coefficient was 0. 578, indicating a statistically significant relationship at the 0.01 level, as shown in Table 6. Thus, the results indicated that the attitudes and perceptions of women toward the process of egg freezing were positive and statistically significant. In other words, the participants with a positive attitude toward voluntary egg freezing were more likely to have positive perceptions of this technique, and vice versa.

**Table 6:** Correlation between women’s attitudes and perceptions regarding oocyte cryopreservation.

Items	Perceptions	
	*r*	*P*-value
Attitudes	0.578**	0.000

**The correlation is significant at the 0.01 level (two-tailed).

Women with higher educational attainment—those with a bachelor’s degree (knowledge = 2.8 ± 0.6, attitudes = 2.4 ± 0.7, perceptions = 2.6 ± 0.6) and a postgraduate degree (knowledge = 3.1 ± 0.5, attitudes = 2.9 ± 0.4, perceptions = 2.9 ± 0.3)—demonstrated significantly better knowledge, more positive attitudes, and more favorable perceptions of oocyte cryopreservation compared to those with only a diploma (knowledge = 2.4 ± 0.7, attitudes = 2.1 ± 0.6, perceptions = 2.3 ± 0.8). These differences were statistically significant (*p* < 0.05). Urban women (knowledge = 2.9 ± 0.6, attitudes = 2.5 ± 0.5, perceptions = 2.7 ± 0.6) scored significantly higher on all three measures than rural women (knowledge = 2.5 ± 0.7, attitudes = 2.2 ± 0.6, perceptions = 2.4 ± 0.7), with *p*-values of 0.031, 0.045, and 0.020, respectively. There were no statistically significant differences in knowledge, attitudes, or perceptions based on marital duration, with *p*-values of 0.264 (knowledge), 0.340 (attitudes), and 0.289 (perceptions) across all duration categories. Women with higher income levels, particularly those who could cover treatment costs and save money (knowledge = 3.0 ± 0.4, attitudes = 2.8 ± 0.3, perceptions = 2.9 ± 0.4), had significantly higher scores than those whose income was not enough to cover treatment costs (knowledge = 2.3 ± 0.6, attitudes = 2.0 ± 0.6, perceptions = 2.2 ± 0.6), with p-values of 0.001, 0.002, and 0.003, respectively (Table 7).

**Table 7:** Statistical differences in knowledge, attitudes, and perceptions by demographic variables (*N* = 202).

Variable	Group	Knowledge (Mean ± SD)	Attitudes (Mean ± SD)	Perceptions (Mean ± SD)	*P*-value (Knowledge)	*P*-value (Attitudes)	*P*-value (Perceptions)
Education level	Diploma	2.4 ± 0.7	2.1 ± 0.6	2.3 ± 0.8	0.002*	0.01*	0.004*
	Bachelor’s	2.8 ± 0.6	2.4 ± 0.7	2.6 ± 0.6			
	Postgraduate	3.1 ± 0.5	2.9 ± 0.4	2.9 ± 0.3			
Residence	Urban	2.9 ± 0.6	2.5 ± 0.5	2.7 ± 0.6	0.031*	0.045*	0.020*
	Rural	2.5 ± 0.7	2.2 ± 0.6	2.4 ± 0.7			
Marital duration	<1–<4 yrs	2.7 ± 0.6	2.3 ± 0.6	2.5 ± 0.7	0.264	0.340	0.289
	5–7 yrs	2.8 ± 0.5	2.4 ± 0.5	2.6 ± 0.6			
	8–10 yrs	2.6 ± 0.6	2.3 ± 0.7	2.5 ± 0.7			
	>10 yrs	2.5 ± 0.7	2.2 ± 0.6	2.4 ± 0.6			
Income level	Not enough	2.3 ± 0.6	2.0 ± 0.6	2.2 ± 0.6	0.001*	0.002*	0.003*
	Enough	2.8 ± 0.5	2.4 ± 0.5	2.6 ± 0.6			
	Enough and able to save	3.0 ± 0.4	2.8 ± 0.3	2.9 ± 0.4			

*p-values were derived from ANOVA or *t*-tests, depending on the variable type. A *p*-value < 0.05 indicates statistical significance.

## Discussion

Oocyte cryopreservation is a rapidly advancing and groundbreaking technique that involves the extraction, freezing, and storage of a woman’s oocytes. The primary objective of this study was to assess the knowledge, attitudes, and perceptions of women with ovarian diseases regarding this technology. The study findings indicated that the majority of the participants held a bachelor’s degree, had been married for 1–4 years, and resided in urban areas. The majority of the participants were between the ages of 20 and 30 years, and less than half had sufficient financial resources to cover their treatment costs. These results align with the findings of Farrag and El-Tohamy (22), who reported that the majority of participants were aged 18–26 years, had a university education, and lived in urban settings. Similarly, El-Adham and Shaban (23) found that the majority of participants were urban dwellers, held university or postgraduate degrees, and had inadequate family income. These findings were also supported by Anaby et al. (24) and Xie et al. (8). A significant number of participants reported that their ovarian disease negatively impacted their overall quality of life. Ligocka et al. (25) noted that, although many women with polycystic ovary syndrome (PCOS) rated their quality of life as excellent or very good, they still found the symptoms of PCOS to be frustrating. Women with lower quality of life scores were more likely to report that PCOS affected their daily lives, made them feel helpless, caused depression, and led to dissatisfaction with their appearance. These findings are supported by the studies conducted by Agrawal et al. (26), Joshi et al. (27), and Saeed Abd Elaziz et al. (28).

PCOS was the most prevalent condition among the participants, with fewer than one-quarter reporting symptoms that lasted for 1–2 years. A similar number of patients had been diagnosed with ovarian diseases within the same timeframe. Additionally, a considerable proportion of women in the study faced infertility challenges as a result of their ovarian conditions. These findings are consistent with those of Al Anwar et al. (29), who noted that young women with PCOS commonly experience infertility, menstrual irregularities, and chronic anovulation. PCOS increases the risk of infertility by as much as tenfold, affecting approximately 40% of those diagnosed with the condition.

The study also revealed substantial gaps in participants’ knowledge regarding oocyte cryopreservation. The majority of women demonstrated an inadequate understanding of the technology. This may be due to its relatively recent introduction, cultural hesitance, and a general preference for conception using fresh oocytes. Farrag and El-Tohamy (22) and Xie et al. (8) similarly reported that the majority of participants had unsatisfactory knowledge of oocyte cryopreservation. Hasab Allah et al. (30) found that over half of the students surveyed had poor knowledge scores. These findings are further supported by Anaby et al. (24), El-Adham and Shaban (23), and Mohamed et al. (1). A study conducted at Islamic Azad University in Tehran by Rafiei et al. (12) also reported poor fertility preservation knowledge among nursing students. Ng et al. (31) found limited awareness of fertility preservation among Chinese medical students. These findings are also supported by the studies of Akhondi et al. (32), Mahesan et al. (11), Mohamed et al. (1), and Zhou et al. (21).

Regarding attitudes, the study found that more than half of the women held negative views toward oocyte cryopreservation. Many strongly believed that paying for this elective procedure was unacceptable, citing the high cost as unaffordable. Similar findings were reported by Hasab Allah et al. (30), who found that nearly three-quarters of their participants held negative beliefs about egg freezing, with only a quarter showing a positive attitude before intervention. However, Xie et al. (8) noted a more positive outlook toward elective oocyte cryopreservation among their participants. Belal et al. (33) also found that fewer than one-quarter of female nursing students held positive attitudes before participating in educational programs. In contrast, Akhondi et al. (32) assessed knowledge and attitudes toward social and medical oocyte cryopreservation and found favorable opinions among female university students in Tehran. Yeung et al. (20) reported that while Hong Kong Chinese individuals had low levels of awareness, their attitudes toward oocyte cryopreservation were generally positive. Zhou et al. (21) also observed that despite having limited knowledge, college students were highly accepting of egg freezing and open to discussing the ethical considerations involved. This openness may be attributed to the better quality of life and greater affordability in more developed nations.

There was a statistically significant relationship between participants’ knowledge, attitudes, and perceptions regarding oocyte cryopreservation. Improved knowledge appeared to enhance attitudes and perceptions toward the practice. These findings highlight the urgent need for health education, counseling programs, and official guidelines on oocyte cryopreservation. Maternity and gynecological nurses should be empowered to provide accurate information to unmarried, healthy women to support informed decision-making regarding fertility preservation (34–37). These findings are consistent with those of Farrag and El-Tohamy (22), who also found a significant correlation between couples’ knowledge and perceptions. Supporting evidence was provided by Belal et al. (33), Hasab Allah et al. (30), Mohamed et al. (1), and Ng et al. (31). The study successfully addressed its research questions and raised important concerns about women’s knowledge, attitudes, and perceptions regarding oocyte cryopreservation. The results underscore a clear need for educational interventions to increase awareness and improve acceptance of fertility preservation methods. Knowledge plays a key role in shaping attitudes and perceptions, and improving women’s understanding of oocyte cryopreservation may lead to better reproductive health outcomes.

However, several limitations must be acknowledged. The use of purposive sampling in a limited geographical setting may affect the generalizability of the results. As the data were self-reported, response bias cannot be ruled out. In addition, the absence of longitudinal follow-up limits the ability to assess changes in knowledge or attitudes over time. Despite these limitations, the research question holds significant value, and conducting further qualitative and quantitative research in this area is highly encouraged.

## Conclusion

Based on the findings of the present study, it can be concluded that the majority of the participating women demonstrated inadequate knowledge and held negative attitudes toward oocyte cryopreservation, which, in turn, influenced their overall perception of this fertility preservation method. In addition, more than one-third of the participants reported experiencing difficulties with conception, and ovarian disease was found to negatively impact the overall quality of life for the majority of the women studied.

## Data Availability

The raw data supporting the conclusions of this article will be made available by the authors without undue reservation.
